# Between Human and Machine: University Teachers’ Accounts of Judgment, Metacognitive Defense, and Cognitive Offloading in Generative AI Use

**DOI:** 10.3390/jintelligence14100254

**Published:** 2026-10-10

**Authors:** Zhen Qiang, Zhuo Wang

**Affiliations:** 1 College of Education, Tianjin University, Tianjin 300350, China; 2 College of Foreign Languages, Lanzhou Jiaotong University, Lanzhou 730070, China; 3 College of Educational Sciences, Qingdao University, Qingdao 266071, China

**Keywords:** cognitive offloading, metacognitive monitoring, professional judgment, teacher beliefs, thematic analysis, university teachers

## Abstract

Generative artificial intelligence (GenAI) allows university teachers to delegate cognitive work while deciding how students should engage with it. Using codebook thematic analysis, we examined multi-source accounts from 25 teachers recruited through an AI-focused learning community and its professional networks in China. Materials comprised interviews with 22 teachers, three documentary cases, self-narratives and reflective essays. Three themes organize the findings: reconstructing the teacher role, defending professional judgment, and managing teachers’ and students’ cognitive participation. Participants described disciplinary expertise as a basis for evaluating AI outputs and reported practices intended to retain human engagement. Instrumental and partnership orientations overlapped; descriptive comparison with later use profiles did not establish a simple relationship between partnership language and usage intensity. Because the protocol introduced several focal topics, we distinguish prompted positions from volunteered formulations. The proposed belief–strategy–practice cycle is a heuristic interpretation of retrospective accounts, not a demonstrated mechanism. The study contributes an account of how teachers connect their own cognitive offloading with their reported organization of students’ learning opportunities. Its claims concern this AI-engaged sample rather than university teachers generally.

## 1. Introduction

Since late 2022, generative artificial intelligence (GenAI) tools such as ChatGPT, DeepSeek, and Doubao have become increasingly visible in university teaching. Students can produce essays and translations with a single prompt; teachers can prepare lessons, design assessments, and give feedback with comparable ease. Beyond efficiency, these capabilities unsettle established assumptions about “knowledge and teaching”. When a machine produces a “well-structured, logically coherent” answer in seconds, questions about teachers’ epistemic authority, professional value, and educational purpose move to the foreground (Bearman & Ajjawi, 2023; Markauskaite et al., 2022).

These changes also raise a question about cognitive offloading: which parts of a task can be delegated without displacing the cognitive engagement that learning requires (Risko & Gilbert, 2016)? Teachers occupy two relevant positions. They may use AI to support their own work, while designing tasks and assessments that influence students’ opportunities to delegate theirs. Understanding how teachers describe this combination connects professional judgment with metacognitive regulation and the conditions for learning. The present study examines these accounts rather than measuring changes in intelligence or learning performance.

Much early research on educational technology adoption has examined whether and how teachers use new tools, often through acceptance models (TAM/UTAUT) or competence frameworks such as TPACK (Al-Abdullatif, 2024; Miranda & Chamorro-Mera, 2026). GenAI, however, raises a different set of questions—“What can AI replace?”, “What is left of me as a teacher?”, and “Why should humans still learn by themselves?”—that concern beliefs and cognition rather than operational skill alone. Teachers’ beliefs about whether AI is a tool, partner, or rival appear to orient how they use it, where they establish boundaries, and how they reposition themselves (Farazouli et al., 2025). Their experiences of sustained interaction with AI—including how they understand, verify, and monitor its outputs—also reveal cognitive dimensions of digital literacy (Sun et al., 2026; Zhou & Schofield, 2024). This study therefore treats belief and cognition as analytically distinct but mutually shaping aspects of teachers’ accounts of professional change.

Accordingly, we ask:

What do university teachers describe believing about GenAI—its nature and boundaries, teaching and the teacher role, and their own professional value?

What cognitive strategies do teachers report using as they work with AI, including how they verify and judge its outputs and how they guard their own and their students’ cognitive participation?

Which formulations recur across the 25 cases, which of them were volunteered rather than elicited by the interview protocol, and how do participants describe the relation between what they believe and how they think?

## 2. Literature Review and Conceptual Framework

### 2.1. Teacher Beliefs and Teacher Cognition

In teacher research, beliefs have long been understood as psychological constructs that are more enduring than knowledge and that filter perception and action. Pajares (1992) argued that teacher beliefs are “the best indicators of the decisions individuals make throughout their lives” in teaching, while Kagan (1992) emphasized that such beliefs are both stable and context-sensitive. The language-teacher cognition tradition (Borg, 2003) brings together what teachers “think, know, and believe” under the broader construct of teacher cognition and emphasizes reciprocal relations among beliefs, knowledge, thought, and practice.

For analytical purposes, we distinguish the two constructs without treating them as independent. Beliefs are teachers’ relatively stable assumptions and value judgments about what the world is and how it should be (for example, “AI is a tool”; “education is life influencing life”). Cognition refers to process-oriented activities of information processing and reflection (mental models of AI, strategies for verifying AI output, metacognitive monitoring, cognitive change). Beliefs orient and filter cognition, while cognitive experience may consolidate or revise beliefs. This reciprocal formulation belongs to the teacher-cognition tradition itself, in which belief, knowledge, thought and practice stand in mutual rather than sequential relation (Borg, 2003); we do not derive it from models of the layered structure of teacher identity, which describe how professional commitments are organized rather than how beliefs and cognition act upon each other.

What it means to extend this relation to GenAI is worth stating precisely, because the extension is what the analysis rests on. We take it to involve three claims. First, GenAI engages beliefs about the nature of knowledge and about how learning happens, not only beliefs about a tool: a system that produces defensible prose on demand bears on what teachers hold about the value of effortful practice. Second, it therefore bears on the teacher’s claim to epistemic standing rather than on adoption alone, since the question is not whether to use the system but what remains distinctively the teacher’s to do. Third, the cognitive work at issue is the appraisal and authentication of machine-produced claims, which is a different activity from the retrieval and explanation that teaching has conventionally required. These three claims are what the coding scheme operationalizes, and Section 2.4 states which belief and cognition domains follow from each.

### 2.2. Technology Integration Research: From External Barriers to Internal Beliefs

Research on educational technology integration commonly distinguishes “first-order” barriers (external factors such as resources, training, and policy) from “second-order” barriers (internal factors such as beliefs, attitudes, and self-efficacy) (Ertmer, 1999). In the GenAI context, many tools are readily accessible and require relatively little setup, making internal factors especially visible even though access and institutional conditions remain uneven (Athanassopoulos et al., 2026; Miranda & Chamorro-Mera, 2026). Competence frameworks have been extended accordingly, from AI literacy for higher education generally (Zhou & Schofield, 2024) to GenAI-responsive competency models for university teachers specifically (Sun et al., 2026) and to the pedagogical, rather than merely technical, dimension of teachers’ AI preparation (C. Wang & Zhu, 2026). Scholarship has framed GenAI both as an “empowering tool” and as an “academic-integrity disaster”, reflecting a wider tension between pedagogical opportunity and integrity concerns (Bearman & Ajjawi, 2023; Bittle & El-Gayar, 2025; Gonsalves, 2025; Perkins, 2023). Teachers’ working assumptions about what AI can and cannot do may therefore shape the depth and form of classroom integration.

### 2.3. New Issues Raised by GenAI for Teacher Identity and Cognition

GenAI foregrounds three issues for teacher research. First, it may destabilize epistemic authority: when AI can answer student questions immediately, the teacher is no longer the sole readily available source of knowledge (Selwyn, 2019; Yu, 2018; Zawacki-Richter et al., 2019). Recent work treats this destabilization as an active negotiation rather than a simple loss, examining how professionals decide when to defer to a machine and when to override it (Haase, 2026; Jose et al., 2025; Milella & Cabitza, 2026). Second, it intensifies debate about cognitive offloading, because the possibility that tools may displace rather than support thinking has become a practical pedagogical concern (Risko & Gilbert, 2016). Empirical studies now report that generative tools can raise task performance while depressing the effortful regulation that underpins learning—what Fan et al. (2025) term “metacognitive laziness”—while observational and review evidence describes variation in associations among AI use, offloading, critical thinking and learner agency (Gerlich, 2025; Si et al., 2026; G. Wang et al., 2026). Third, it creates a new cognitive division of labor in human–AI collaboration. Practices that keep a “human-in-the-loop”, together with prompt design and output verification, point to a form of digital literacy centered on teachers’ capacity to manage machine-generated content through professional judgment (Essien et al., 2026; Yang et al., 2024).

Empirical work on university teachers themselves is growing but remains uneven. Survey and Q-methodological studies map perceptions and acceptance (Al-Abdullatif, 2024; Espartinez, 2024; Miranda & Chamorro-Mera, 2026); qualitative studies describe uncertainty and improvisation in the first years of GenAI-affected teaching (Farazouli et al., 2025); reviews call for teacher development to move beyond tool training (Panday-Shukla, 2025; Tan, 2025). Two gaps persist. Most studies examine perceptions at a single point rather than the reciprocal traffic between what teachers believe about AI and how they think while using it, and most sample pre-service or single-discipline teachers rather than experienced academics across disciplines. Against this background, the present study examines how teachers describe that traffic and asks which formulations recur across a multidisciplinary sample.

Research on preservice teachers also connects beliefs about GenAI with intentions for practice (Karlin et al., 2026). Here, the focus is on experienced teachers’ descriptions of their professional work, with attention to differences among cases and to the limits of retrospective accounts.

### 2.4. Professional Identity and Agency as the Frame for Judgment and Responsibility

The questions this study raises about judgment, responsibility and professional value are questions about professional identity, and they are better served by that literature than by a general account of belief. Beijaard et al. (2004) describe professional identity as an ongoing process in which teachers interpret and reinterpret their experience, constituted in relation to context and sustained through the stories teachers tell about what their work is for. This is the vocabulary that fits an occupational group whose members are renaming their role while the conditions of the work change around them, and it treats such renaming as identity work rather than as attitude change.

Agency supplies the second term. Biesta et al. (2015) argue that agency is achieved rather than possessed, arising from the interaction between a teacher’s repertoire and the conditions of a given situation, and that beliefs function within it as a resource teachers draw upon rather than as a private mental state that issues in behavior. Two consequences matter here. Beliefs that enable action in one situation may constrain it in another, so a teacher’s conviction about what machines cannot do may support principled refusal in one setting and unexamined avoidance in another. And because agency is achieved in context, appeals to agency of the kind our participants make are claims about the conditions under which they can act, not assertions of individual willpower.

These two sources also fix the relation between the framework and the coding scheme. The belief domains in the codebook follow from the first claim in Section 2.1 (the nature and boundaries of AI, teaching, self and value); the identity codes follow from Beijaard et al.’s account of renaming and reinterpretation; the cognition domains follow from the third claim (mental models, verification and judgment, metacognitive monitoring, cognitive change); and the codes concerning defense and boundary-setting follow from Biesta et al.’s treatment of beliefs as resources for achieving agency. Table 1 records the assignment of every code to belief, cognition, identity, affect or practice, together with the rule applied when a code spanned more than one construct.

## 3. Methods

### 3.1. Design and Participants

We adopted a basic qualitative design (Merriam, 2009), comparing across cases to examine both commonality and variation in teachers’ accounts. The analytic corpus comprises 25 teacher cases (Table 2). Of the 25, 22 took part in semi-structured interviews; for the remaining three (Teachers D, E, and F), no interview was conducted, and their case materials consist of field notes together with, respectively, chat records, product documents, and published essays. The first author’s reflective texts are not part of the coded corpus and contribute no evidence to the analytic case counts, coverage figures or stance distributions; they are used as reflexive material and are described in Section 3.5.

Twenty-two participants are women and three are men. They were aged between 33 and 60 (*Mdn* = 43) and had between 7 and 30 years of teaching experience (*Mdn* = 16); ranks ranged from lecturer to professor, and one participant had recently retired. Their disciplines were foreign languages and applied linguistics (10, including College English, English for academic and medical purposes, business and international communication English, teacher training, and Japanese), journalism, communication and advertising (3), art and design (2), law and political science (2), educational technology and information technology (2), and one each in ideological and political education, medical psychology, mental health education, management science and engineering, logistics and supply chain management, and electronic commerce.

The 25 teachers work in 13 provinces and municipalities, with six in Gansu and three in Guangdong and the remainder distributed singly or in pairs across Shaanxi, Hunan, Hebei, Guizhou, Guangxi, Chongqing, Shandong, Henan, Yunnan, Jiangsu and Zhejiang. Their institutions comprise one university in the 985, 211 and Double First-Class schemes, two in the 211 and Double First-Class schemes, six provincial key universities outside those schemes, two normal universities, two medical universities, three vocational colleges, and one each of a foreign-language university, a university for nationalities, a university of political science and law, a university of Chinese medicine, a science and engineering university, a Sino-foreign cooperative institution, an independent undergraduate college, a provincial comprehensive university and a provincial undergraduate institution. The participants therefore work in a varied set of institutions that extends well beyond the elite tier. Because the sample was assembled through purposive and snowball sampling within a single learning community, it is not representative of Chinese higher education, and this institutional variety describes the settings of the accounts without supporting generalization to the sector.

Participants were recruited through purposive and snowball sampling within a learning community devoted to AI-empowered teaching, to which the first author belongs. The community was founded in December 2023, with the purpose of discussing and sharing emerging GenAI tools and creative uses among university teachers and doctoral students. Recruitment targeted university teachers in mainland China with sustained use of GenAI in professional work; the included sample also contained one recently retired teacher, whose status is reported explicitly; no criterion concerning attitude towards AI was applied, although the recruitment route makes committed users more likely to be included. Recruitment proceeded from community members known to the first author to teachers they nominated in turn.

The composition of the sample is a property of the study rather than an incidental feature. Foreign-language teachers make up 10 of the 25 cases, and every participant was already engaged with GenAI at the time of recruitment (though with varied levels of use or intensity). Teachers who had considered GenAI and rejected it, and teachers who had never encountered it, are not represented.

This was an insider study: the first author and most participants belong to the same learning community that was open to rolling recruitment, and some interviews and self-narratives were produced during community events. This position afforded trust, contextual understanding and access to naturalistic accounts. It also shapes what participants chose to disclose and how their accounts were interpreted, and it means that a shared way of talking about AI is available as an explanation for part of what we report as convergence.

### 3.2. Data Collection

Materials were collected between January 2025 and May 2026. Twenty-nine people were approached and four declined participation, leaving 25 teacher cases. Of these, 22 contributed semi-structured interviews and three (D, E and F) contributed documentary material. Three source types anchored the corpus, supplemented by other materials made available by individual participants:

Semi-structured interviews (22 teachers, 60–120 min each) followed a common revised protocol, Exploring University Teachers’ Roles and Digital Literacy in the GenAI Era. The protocol comprised six sections: background and first contact with AI; digital literacy and technology adaptation; teaching roles and experiences; digital ethics and critical AI engagement; hypothetical and critical thinking; and reflective and future-oriented questions. Because several sections put propositions to participants, Table 3 records which topics the protocol introduced and which formulations participants volunteered. The interviewer’s field notes supplemented the transcripts. Teachers J and W each took part in more than one interview, and Teacher R also completed the protocol in written form.

All interviews were conducted, recorded and transcribed by the first author on Tencent Meeting, the videoconferencing platform on which the interviews took place. The platform exports two forms of the same recording: a verbatim export and a cleaned export that it labels “intelligent-optimized”. The cleaning is performed automatically by the platform as part of the export. For each interview, a single transcript was manually checked and corrected against the recording before being used for coding. No separate generative-AI system was used to rewrite the transcript texts. The original recordings were retained by the first author for checking the transcript and quotations. The first author stored the original recordings and transcripts on a password-protected personal laptop; access to these original files was limited to the first author. Tencent Meeting processed recordings containing participants’ names. The first author removed names and identifying details from the transcripts before sharing deidentified copies with the corresponding author.

Three interviews were held in both forms, which allows the effect of the cleaning to be measured rather than assumed. The cleaned export is 9 to 11 per cent shorter than the verbatim export and agrees with it at the character level between 0.83 and 0.94. Most of the difference consists of discourse particles, false starts, repetitions, laughter markers and opening exchanges about the recording. The comparison also shows that the cleaning occasionally substitutes a word rather than deleting it, and we report this rather than treating the two forms as equivalent. For one case, the cleaned export carries speaker attribution that the verbatim export lacks, and it was retained for that reason. Because the cleaning can alter wording, every quotation reproduced below has been checked against the verbatim export or, where only the cleaned export exists, against the retained recording; quotations are taken from the verbatim text. Table 2 records the transcript forms identified in the source inventory; an unlabeled form is not assumed to be verbatim. Additional exports retained for comparison were not treated as additional cases or independently coded versions. The technical version of the platform’s export facility is not specified in the retained source descriptions.

Self-narratives comprised 11 ten-minute presentations from 10 teacher participants, delivered at a community gathering on 26 January 2025. The number of presentations therefore differs from the number of contributing cases. Table 2 identifies the ten cases with narrative material in the analytic inventory. The first author’s presentation is excluded from these counts and treated separately as reflexive material.

Reflective and documentary texts included participants’ published WeChat public-account essays (Teachers F, L, and O) and the first author’s reflections. For the three cases in which no interview took place (Teachers D, E, and F), the analysis rested on field notes together with, respectively, chat records, product documents, and published essays; Teacher H additionally supplied recorded rubric-design dialogues with AI. These materials are listed case by case in Table 2.

During revision, supplementary participant information was assembled on commonly used tools, frequency, approximate hours per day, purposes and use in work with students. Table 2 reports tools and usage intensity; Supplementary Table S1a reports the purposes recorded in the participant information sheet. These profiles describe use reported during revision and are distinguished from the earlier interview accounts.

The revision-stage profiles and interview materials differ in timing and in the questions used to elicit tool use. The profiles therefore provide descriptive context, rather than a reconstruction of the circumstances of every earlier account. Section 4.2.2 compares these profiles with the partnership classification without inferring that later usage produced earlier beliefs. Tool-specific turnover rates are not estimated because the two sets of reports are not equivalent inventories.

All 25 participants were reported to use GenAI in work with students, but this broad designation includes different activities, from preparing materials for students to organizing learning tasks and supporting research. It does not imply that all participants allowed students direct, unrestricted access to AI. The purposes recorded case by case are summarized in Supplementary Table S1a; where the profile does not specify an activity, none is inferred.

The source types served complementary analytic functions: interviews captured “in-the-moment responses,” self-narratives provided “reconstructed stories,” and essays and other documents represented “sedimented reflection.” Because the cases rest on uneven combinations of these materials, verbs of attribution are used consistently below to mark provenance: “said,” “explained,” and “stated” introduce spoken material, while “wrote” introduces written material.

### 3.3. Data Analysis

We analyzed the corpus through codebook thematic analysis. This variant develops themes interpretively while using a structured codebook to support consistent application across a corpus larger than a single analyst can hold in view (Braun & Clarke, 2021). Codebook approaches do not require a measure of intercoder agreement, and such measures are not a criterion of analytic quality in this tradition. We used Cohen’s *κ* in a limited, supplementary role, as a check on whether the code definitions could be applied consistently by a second coder, and relied on a code-by-case matrix to keep the evidence for each theme traceable. These procedures distinguish the analysis from reflexive thematic analysis, which treats analyst subjectivity as a resource and does not use agreement measures. Coding combined deductive and inductive logics. Deductively, the framework set out in Section 2.1 and Section 2.4 supplied the belief and cognition domains: belief domains concerned AI’s nature and boundaries, teaching, self and identity, and values; cognition domains concerned mental models, learning processes, verification and judgment, metacognitive defense, cognitive conflict, and cognitive change. Inductively, we remained open to emic concepts (baguan [gatekeeping], xinli [inner strength], “doing raises problems, thinking is the answer”).

Analysis proceeded through three rounds. First, the first author coded the corpus segment by segment and prepared analytic memos in six working groups used to organize the materials, not as theoretical or demographic categories. Second, initial codes and memos were consolidated into Codebook V1.0, with 20 codes retaining the identifiers B1–B11 and C1–C9. Definitions, inclusion/exclusion criteria and the recorded boundary refinements are provided in Supplementary Section S3; Table 1 distinguishes their primary and secondary constructs. Third, the first and corresponding authors independently assigned primary codes to 45 meaning units from 19 teacher participants and one first-author reflective source, covering all six working groups and the 20-code scheme. Agreement was 88.9% (40/45), with Cohen’s *κ* = 0.881. Excluding the first-author unit (S44) leaves 44 teacher-derived units, with 88.6% agreement (39/44) and *κ* = 0.878. The five disagreements concerned primary-code boundaries and were reviewed against the original texts, with final adjudication by the corresponding author; the codebook was revised to V1.1. Only the first author belonged to the learning community. The coefficients describe consistency in primary-code assignment on this subsample only; they do not cover the separate case-level evidence-strength ratings and do not by themselves establish the quality of the themes.

Coding was conducted on the Mandarin Chinese originals. The corresponding author translated quotations into English and checked the English wording after coding. No additional bilingual researcher independently checked the translations. Culturally specific terms are romanized and glossed at first use, and the Mandarin source remains the basis for interpreting their meaning. The absence of an additional bilingual check is a limitation of the English presentation.

We then constructed a code-by-case matrix (25 × 20; Supplementary Table S2a,b, summarized in Appendix D) to trace the evidence supporting candidate themes. Each cell records clear evidence (an explicit statement of the proposition in the participant’s own words), partial evidence (material that implies the proposition or addresses it obliquely), a counter-case (an explicit statement incompatible with it), or no evidence. These evidence-strength ratings are a separate decision from the primary-code assignments assessed by κ. An absent code records the absence of evidence in the materials available for that case rather than the absence of the belief, and the three cases documented without an interview are the most affected. To make the evidentiary basis of each candidate theme explicit, we adopted a reporting convention under which a formulation was treated as widely shared when clear or partial evidence appeared in at least three-quarters of cases and clear evidence in at least one third. This convention was adopted after the matrix had been compiled and inspected, because coverage alone did not discriminate among codes once most proved to be broadly distributed; it is a device for reporting rather than a test the themes passed, and Table 4 reports how the set of qualifying codes changes under stricter rules and alternative case definitions. Two qualifications follow. The survival-belief code B7 reaches the coverage level (23/25) but not the clear-evidence level, with clear evidence in six cases under every rule examined; the filter-and-polarization vocabulary is therefore reported as a shared idiom rather than as a firmly evidenced belief. The partnership code B2 (10/25) falls below the coverage level and is reported as a point of divergence. We also note that several code definitions are broad enough that near-universal coverage was to be expected; coverage is therefore never reported on its own, but alongside the distribution of clear evidence and the code’s prompted status.

Because the protocol put several of the propositions at issue to participants directly, we audited it against the codebook and classified each code by whether the protocol introduced the topic (Table 3, with the protocol wording quoted). Seven codes are prompted: the “intellectual degeneration” proposition was supplied verbatim and participants were invited to agree or disagree; the boundary codes follow from a question about what AI cannot replace; the epistemic-authority codes follow from the “content deliverer” critique; and the verification code follows from a direct question about verification. Eight codes are framed, in that the protocol opened a topic without supplying the position or the formulation participants produced. Five codes are unprompted: no protocol item introduces the partnership orientation, the filter-and-polarization idiom, the metaphors of how AI works, the efficiency–depth tension, or the learning community. Where a theme rests on prompted material, the findings report the distribution of positions taken towards a proposition we supplied and do not treat recurrence as evidence of a spontaneously shared orientation.

### 3.4. Trustworthiness

Credibility was supported by triangulation across source types, an audit trail (codebook, coding matrix, stance classifications, and per-case evidence files), and negative-case analysis. Two counter-cases associated with Teacher A—one against the emotion-and-embodiment boundary (B3) and one against irreplaceability construction (B8)—are reported in the findings and recorded in Appendix D. Triangulation operated across source types at the level of the corpus; the availability of multiple source types within a case creates an opportunity for corroboration but does not itself establish agreement among those sources, and claims resting substantially on thinly documented cases are identified as such. The agreement coefficient reported in Section 3.3 is evidence about the communicability of the codebook and not a validity test of the interpretation (O’Connor & Joffe, 2020). All quotations were checked against the verbatim transcripts or the recordings before being reproduced.

The first author’s insider position supported access and contextual understanding, while also creating a risk of shared assumptions. The corresponding author was not a member of the community. Independent coding and review of disagreements added a second interpretation, but neither the agreement coefficient nor final adjudication eliminates interpretive dependence on the researchers’ perspectives.

### 3.5. The First Author’s Material as a Reflexive Strand

The first author is a university teacher in the same community as the participants and has kept reflective writing about working with GenAI over the same period. That material was generated under conditions no participant shared: it was not elicited by the protocol, and its author had access to the full corpus and to the developing analysis. It is therefore not treated as a case. It contributes no evidence to the 25-case matrix, coverage figures, stance counts or substantive findings. One first-author unit had been included in the original codebook consistency exercise; Appendix C reports that exercise transparently alongside the recalculation excluding that unit.

Its function is reflexive. The first author’s own movement from enthusiasm through disappointment to a qualified working relationship with these systems, and the questions that movement raised—what teaching is for when explanation is cheap, and where a teacher’s value then lies—shaped which questions the protocol asked and which formulations were recognized as significant during coding. Recording this is a way of making that influence inspectable rather than of licensing it: analytic memos were used to revisit readings that matched the first author’s own experience too readily, and the negative cases reported below were sought deliberately for that reason.

## 4. Findings

Across the 25 cases, participants returned to a set of concepts when discussing what AI is, what it cannot do, and what they should do about it. Recurrence is interpreted alongside the distribution of clear and partial evidence, protocol prompting and case context. Appendix D summarizes coverage and Supplementary Table S2a,b provide the case-level matrix. Section 4.1 addresses beliefs and professional value (RQ1); Section 4.2 and Section 4.3 address reported cognitive strategies and participation (RQ2); recurring formulations, divergence and retrospective change across these sections inform RQ3. The heuristic synthesis follows in Section 5.4.

### 4.1. Redescribing What a Teacher Is for

This theme draws on the identity codes and on the boundary codes that support them. Its evidentiary basis is broad: clear or partial evidence for role reconstruction appears in every case, and for the two boundary codes in 24 and 25 of the 25 cases (Appendix D). Two qualifications govern how that breadth should be taken. The boundary codes are prompted: participants were asked what AI cannot replace, and what follows is the shape of their answers to that question rather than a preoccupation they raised unbidden. Role reconstruction is framed rather than prompted: the protocol asked about roles, but the renamings—passenger seat, gatekeeper, designer, goalie—were volunteered.

#### 4.1.1. What AI Cannot Do: The Boundary Participants Draw

Participants described AI’s capability boundary with notable consistency, organizing it around five recurring concepts: emotion, embodied experience, metacognition, tacit knowledge, and judgment. The question that elicited these accounts asked what AI cannot replace, and the consistency reported here is therefore a consistency in how participants answered it.

Emotion and warmth constituted the most widely shared boundary. Teacher O (nearly 30 years of teaching) said: “As for emotional value and warmth—it is still a machine you are facing.” Teacher R (business English at a higher-vocational college) grounded this boundary in classroom experience: “Students like teachers who feel ‘alive’—teachers who act cute in class, get angry, lose their temper, and praise them generously.” Teacher U (ideological education) stressed “emotional resonance… empathy”; Teacher Y (Japanese) positioned herself as “an emotional companion”; Teacher W (oil painting) asked rhetorically: “When you talk with a person, can’t you feel their emotion from a single glance?” Teacher I framed the boundary through the classical triad of teaching: “Of ‘transmitting the Way, imparting knowledge, and resolving doubts’ (chuandao shouye jiehuo), it is the last—resolving doubts—that is hard to integrate [with AI]… it requires a certain emotion, or what we call emotional value and offline organization.”

One case ran against this consensus. Teacher A located warmth on the machine’s side of the line, describing AI as able to “transmit energy” and as one that “understands human nature too well”—the single counter-case to B3 in the coding matrix (Appendix D). This account is examined further in Section 4.1.3 and Section 4.2.2, where the same participant also declines the premise of the irreplaceability question.

A second boundary concerned embodied experience and bodily memory. Teacher F, reflecting on a film that rejected AI face-swapping, wrote: “AI face-swapping lacks ‘embodied cognition’—it has no real body… it can only simulate appearances on the basis of data.” Teacher N observed: “The biggest difference between humans and large models now is the connection with nature… it is reflected precisely in the process of collecting data in the field.”

A third boundary centered on metacognition and reflection. Teacher L wrote: “Although current AI can simulate reasoning processes, it has no self-aware subject engaged in reflection. It does not know what it knows, still less what it does not know.” She summarized this as “machines have no metacognition,” arguing that “this deep, self-conscious reflection is a uniquely human, extremely slow background program.”

Tacit knowledge constituted a fourth boundary. Teacher L invoked Polanyi’s paradox—“we know more than we can tell”—arguing that the sense of balance in cycling or the perception of atmosphere in negotiation “cannot be encoded into explicit language or formulas, and so cannot easily be learned by AI.” Teacher V developed this point pedagogically: teachers’ value lies precisely in “letting students see the process by which tacit knowledge becomes explicit… there is a human factor in it.”

The fifth boundary—judgment—was most directly connected to the teaching profession. Teacher B explained: “What probably cannot be replaced is our judgment… what is true and reliable, judged critically.” Teacher G similarly stated: “I would still say judgment… the teacher’s part is to guide students to ask good questions… and to judge the answers.”

We interpret this boundary not simply as a passively accepted “technological fact” but as an actively constructed belief with two related functions: it helped participants make sense of technological capabilities and establish a psychological defense line for professional identity (Section 4.1.3).

#### 4.1.2. From “Knowledge Porters” to Designers, Guides and Companions

The destabilization of epistemic authority was accompanied by a widespread renaming of the teacher role, evidenced in every case. Fifteen of the 25 cases (60.0%) no longer treated “knowledge transmitter/knowledge expert” as their primary self-positioning, instead using terms such as guide, designer, organizer, coach, companion, gatekeeper, navigator, goalie, and trainer. These terms were volunteered rather than offered by the protocol.

Participants first questioned “transmission” itself. Teacher C (more than two decades) described himself self-critically as a “porter of word meanings”: “We are porters at best… I retell things to students; call it a porter, because I really have nothing original.” Teacher J offered a similar assessment: “We are just porters of knowledge… those experts who publish papers, they create knowledge, they create new knowledge.” Teacher B said: “I am not the only authority”; Teacher P: “A teacher’s knowledge is not necessarily authoritative. AI’s knowledge is clearly stronger than mine.”

Participants described this reconstruction along four overlapping paths:

The designer pathway. Teacher V moved “from knowledge-dominated [teaching] to a designer and guide of instruction”, describing the task as setting work that cannot be completed with a single prompt. Teacher L developed this pathway to its extreme: “The right way to open an agent is not to become a skilled tool user, but a deep-thinking systems designer.”

The companion pathway. Teacher R used a representative metaphor: “We used to be the ones driving the car, taking students from start to finish. But now every student has their own car; we have to give up the driver’s seat and sit in the passenger seat to accompany them.” She went on to define herself as a “trainer”: “At university, the teacher is really a trainer—I train you how to learn, how to solve problems.”

The coach/gatekeeper pathway. Teacher D gave three words: “integrator of knowledge, a coach, a supervisor of learning”; Teacher M: “empowerer, guide, gatekeeper (of ethics and truth)”; Teacher L: “I have to take responsibility for what I say… my knowledge base really cannot compare with AI’s, but I think we can play the role of gatekeeper.”

The empowerment pathway. Teacher P adapted “empowerment-based education” from her gender-studies background into an “empowering lecturer” position: “sharing information, promoting thinking, respecting decisions.”

Teacher K condensed this orientation in one sentence: “The matter of ‘being learned’ can be handed over to AI… teachers should be the shapers of students’ souls… what we teachers can do is to model integrity and hand ‘being learned’ over to AI.” Her formulation cedes “being learned enough to be a teacher” (xuegao)—understood as epistemic authority—to AI while retaining “being upright enough to be a model” (shenzheng)—personhood, values, and modeling—as the teacher’s responsibility. This is identity work in Beijaard et al.’s (2004) sense: the occupational category is retained and its content reinterpreted.

This pattern was not uniform: ten of the 25 teacher cases (40.0%) continued to claim a “knowledge transmitter/knowledge expert” positioning (Teachers D, H, J, K, P, Q, S, T, U, Y), although most reinterpreted its content. Teacher K admitted she is currently “still a knowledge transmitter,” adding “I don’t see myself as that kind of guide”; Teacher J insisted “imparting knowledge… moving what I have learned into students’ minds—that has not changed”; Teacher U positioned herself as “a transmitter of knowledge and, at the same time, a guide of values”; Teacher T held that “a teacher is both a learner and a transmitter of knowledge… you should first become an active learner”; Teacher D argued “knowledge expert” and “content deliverer” are “an A+B relation,” not either/or; Teacher P claimed expertise on the ground of professional practice; Teacher H insisted “teachers are still knowledge experts… knowledge has a certain systematic structure”; Teacher S responded: “If you are only a content deliverer, can you do it better than Metaso?”; Teacher Y is “striving to transform into a knowledge expert plus a guide”; Teacher Q offered a reversal: “AI can only take the knowledge from materials it can find, organize it… and deliver it to students… the real production of knowledge lies with humans,” locating the value of academics in “the production of knowledge through research.” Thus, even as epistemic authority was being renegotiated, “knowledge expert” was reinterpreted as “producer, systematizer, gatekeeper, and critic of knowledge” rather than “reciter of knowledge.”

#### 4.1.3. Irreplaceability, and the Idiom of the Filter

When discussing the possibility of being “replaced by AI”, participants drew on structurally similar security narratives. The material is mixed in status: irreplaceability was prompted, since the protocol asked what makes teachers distinctively valuable, whereas the filter-and-polarization vocabulary was volunteered. That vocabulary is reported here as a shared idiom rather than as a firmly evidenced belief, because clear evidence for it appears in only six of the 25 cases (Section 3.3; Appendix D).

The first element of this narrative was the filter metaphor. Teacher F wrote: “AI is not a ‘career terminator’ but a ‘career filter’—it filters out those who have no accumulated depth and can only repeat routine labor.” This formulation parallels the claim Teacher G heard in a Tsinghua training program: “70% of teachers will be eliminated… it is not AI that eliminates people; it eliminates those who cannot use AI.” Teacher V’s version invoked the Matthew effect: “AI has become a Matthew effect—the strong get stronger and the weak stay weak… without this awareness… you will be deprived and replaced.” Teacher Y anticipated “polarization”: “It may replace some; some will become superhuman.”

A second element involved constructing irreplaceability by turning the boundary checklist in Section 4.1.1 into a professional defense: AI has no emotion, so the “transmitter of warmth” (Teacher F) is irreplaceable; AI has no embodied experience, so “the aesthetic sensibility I have accumulated is welded onto me; AI cannot take it away” (Teacher P); AI has no judgment, so the “gatekeeper” is irreplaceable; AI has no metacognition, so the “reflector” is irreplaceable. Teacher R also cited the founder of OpenAI, summarizing human advantages as three quotients: “learning quotient (meta-learning), resilience quotient, and emotional quotient.”

A third element was divergence in felt security: despite a shared vocabulary, participants expressed markedly different levels of reassurance and anxiety. Teacher J expressed pronounced anxiety—he had experienced GPT’s translation of Niushi “crushing” his own in class: “I told my students: classmates, we are going to be replaced”; his confession was, “It was real dread… the knowledge you toiled for years to learn is worth nothing in front of AI,” and his strategy, “If you can’t beat them, join them… since we don’t know it, we learn it.” Teacher C spoke of his “rice bowl” and admitted, “I used to be confident that I was an educator. Now I dare not say so.” Teacher D rehearsed moving “from front stage to behind the scenes… becoming AI’s assistant.” Teacher R’s starting point was “my students are about to lose their jobs, and if they lose their jobs, my own rice bowl is in danger,” but later concluded “from initial doubt and panic to a firm conviction now: absolutely not [replaceable]”—because “the more you use it, the clearer its strengths and weaknesses become.” Teacher A (the single counter-case to B8 in Appendix D) challenged the premise: “A person does not have to be valuable… whether one has value is something one decides for oneself.” Teacher S took a harder line: “If your value is so easily destroyed, it shows you may not have had much academic value.” Teacher M placed the discussion in a longer historical frame with the history of technology: “Generation after generation of technology… there is only one law: hope, hype, disappointment… the so-called democratization of technology may be an illusion.”

We therefore interpret the consistency of the boundary checklist in Section 4.1.1 as serving an additional, self-protective function. Delineating the realm “AI cannot do” also delineated, for many participants, the realm “where I am irreplaceable”—a framing through which beliefs about AI’s limits contributed to the reconstruction of professional identity.

### 4.2. Judgment as the Defended Core of Professional Work

This theme rests on the verification code, which carries clear evidence in 24 of the 25 cases, and on the asymmetry between the instrumental and partnership orientations (Appendix D). The verification code is prompted—the protocol asked directly how participants check what AI produces—so the recurrence of verification talk is not itself a finding; what participants say the checking rests on, and what they call themselves when they do it, is. The partnership orientation is unprompted: no protocol item offers the term.

#### 4.2.1. The Gatekeeper: Professional Expertise as the Filter

As AI made content production less scarce, verification, screening, and judgment moved to the center of participants’ accounts of their cognitive work. Across cases, a common gatekeeping logic emerged: professional expertise served as the primary filter, while instrumental checks provided additional support.

First, at least ten participants explicitly described professional expertise as the “foundation” for verifying AI output (C, I, J, M, O, P, S, T, U, X). Teacher O stated: “It rests on your professional attainment—the deeper your foundation, the better you handle it.” Teacher S: “Your own ability has to be there before you can control it; if your ability is not there, you really cannot keep it in check.” Teacher J similarly explained that verification means “judging, on the basis of your own established knowledge, whether it fits your purpose.” Teacher I summarized the basis of his judgment as “my own way of thinking and my own knowledge background. Only these two.”

Second, participants described five complementary verification strategies:

Cross-checking outputs across multiple models (5 explicit adopters: D, F, H, T, V). Teacher D: “When you can’t tell right from wrong, try several different large models.” Teacher V: “cross-use several large models… verification-type models… to verify its sources.” Teacher F had “Doubao, DeepSeek, and Kimi fight each other… Kimi plays the role of a Socrates.” Teacher H used “DeepSeek, Tencent Yuanbao, Doubao, GPT—all of them,” finding that “GPT at first did not spot the error, but the moment I sent it to Doubao, Doubao immediately said there was a mistake.” Teacher T: “search on this platform and that platform; if they all say roughly the same thing, I am more inclined to accept it.”

Consulting authoritative sources (4 explicit adopters: H, K, M, T). Teacher M treated “works published before 2023” as the trust boundary (“after 2023, even papers—I don’t recommend them much, because too much is generated”); Teacher H compared AI output “with classic works in education… to decide what can be absorbed”; Teacher T “looks things up in dictionaries”; Teacher K verified every AI-suggested theory “again on CNKI or Google Scholar.”

Interrogating outputs through follow-up questions (2 explicit adopters: K, M). Teacher M described a strategy of “exhaustive questioning”: “note down everything you don’t understand… then ask back in a structured way what each thing is and what it means”; she urged “daring to question, daring to battle it,” because AI has a “people-pleasing personality.” Teacher K likewise observed the model’s “flattery”—“it agrees with you first and only then offers small suggestions”—and consequently asked students to “refute first, then examine”: “whatever the model says, the first thing is to rebut it… find the problems first.”

Constraining the corpus through knowledge bases (3 explicit adopters: G, Q, S). Teachers Q, S, and G used knowledge bases to keep AI outputs within a defined evidentiary boundary. Teacher S: “It must be based on a bounded range, within the range of correct texts… at least it won’t talk serious-looking nonsense.”

Testing outputs through implementation (2 explicit adopters: E, W). Teachers E and W both emphasized that “only what gets made counts.” Teacher W said AI’s value is that “it did the trial-and-error for me,” but “in the end, students’ creative ability… has to be tested through practice before a result is reached”; Teacher E: “AI-generated things are sometimes perfect… but what is perfect is not necessarily what can be implemented.”

Third, gatekeeping extended from AI outputs to students’ use of AI. Facing AI-generated student assignments, participants commonly sought to make students’ cognitive processes visible: Teacher T limited time in class and collected phones to force students to “complete it independently first”; Teacher R: “my way: make students explain their answers”; Teacher P examined “how much disciplinary knowledge is inside the structured prompt”; Teacher M anticipated that, in future assessment, “the result’s power to explain your ability is almost zero… the future, I think, is the oral defense.”

Taken together, the “gatekeeper” was not merely one participant’s preferred label. It captured a recurring pattern in participants’ accounts of cognitive activity: AI “generates”; humans “judge.” Within this sample, this division of labor became an important anchor for how teachers reconstructed professionalism in the AI era.

#### 4.2.2. Instrumental and Partnership Stances

On the foundational question of “what is AI?”, responses were asymmetrical: an instrumental formulation appears in every case, whereas a partnership formulation appears in 10 of the 25 (seven with clear evidence—C, D, L, N, O, P, Y—and three partial—A, H, I; Appendix D). The partnership orientation is one of the five codes no protocol item introduces. Because every case contains an instrumental formulation, the partnership cases are a subset of the corpus rather than a mutually exclusive group.

The instrumental orientation emphasized AI’s subordinate status: “It is just a tool—it is the computer of its day… there is no need to treat AI as a flood or a beast” (Teacher F); “AI is only a tool, and a tool depends on human initiative” (Teacher P); “it is instrumental by nature” (Teacher W); “remember, it is a mirror. Don’t expect it to be powerful. What it reflects is us” (Teacher M). Teacher K’s version was tool-neutrality: “A tool is neither good nor bad; it depends on the person using it… whether it is used for good.” Accounts associated with instrumentalism emphasized task-centered use, strong insistence on human control (“it is still my assistant; it cannot control me,” Teacher X), and watchful distance—Teacher H’s “from perfect companion to Waterloo” disenchantment with DeepSeek was consistent with this stance.

By contrast, the partnership orientation attributed greater agency to AI: “treat AI as a conversational partner, not merely a tool” (Teacher L); “AI is no longer just a tool; it has become a companion on the road of growth” (Teacher O); Teacher C called AI “friend and companion”; Teacher N described “a collaborator, a partnership, a colleague relationship, a comrade-in-arms relationship”; Teacher Y called AI “a very good partner”; Teacher D: “I treat it as a ‘person’… I am the one who guards the gate, refining and distilling on its basis”; Teacher P: “I have been raising my GPT for more than two years. It has memory.” Accounts associated with partnership emphasized sustained, personified collaboration (AI companions, raising agents), emotional investment (Teacher A: “I often cry when chatting with [the AI]… she understands human nature too well”), and stronger learning commitment (Teacher L wrote “over a hundred AI interaction diaries” in half a year). Teacher I offered a meta-level answer that located the distinction in users’ positioning: “It depends on how people position AI—as a purely instrumental tool or as a partner in collaboration.”

The orientations also appeared in how participants responded to AI’s flattery. Teacher M noted that AI “only says what you want to hear… it is a people-pleasing personality”; Teacher D’s distaste for “rainbow-fart” praise led him to ask for a more critical exchange—“what you want to hear is some critical content, the good along with the bad.” Whether teachers can retrain AI from “flatterer” into “critic” served in the data as one indicator of increasingly critical engagement.

These orientations were neither mutually exclusive nor static. Several participants described movement “from tool to partner” (Section 4.3.3), while others, including Teacher C, moved among “helper, companion, teacher” positions. Comparison with the revision-stage profiles does not indicate a simple intensity gradient. The ten partnership-coded cases reported 1–3 h of use per day (median 2), compared with 1–4 h (median 2) in the other fifteen cases. Seven of the ten partnership cases and twelve of the other fifteen reported daily or almost-daily use. Partnership formulations occurred in language-related cases (C, D, H, O, Y) and in medical psychology, e-commerce, management, advertising and art (A, I, L, N, P); language teachers also appeared among the remaining cases. These descriptive comparisons indicate that frequency, duration and disciplinary location alone do not distinguish the formulations. They do not estimate an association between contemporaneous use and belief, because the profiles were collected later and the cases are unevenly documented.

### 4.3. Managing Cognitive Participation on Both Sides of the Desk

This theme concerns what participants describe doing about their own and their students’ cognitive engagement. Clear or partial evidence for the defense, conflict and change codes appears in all 25 cases (Appendix D). For cognitive defense, however, only nine cases contain clear evidence and sixteen contain partial evidence; broad category coverage does not mean that every teacher explicitly described each strategy. The status of the material is mixed and is marked in each subsection: the “intellectual degeneration” proposition was supplied verbatim by the protocol, so what follows is a distribution of positions towards a claim we introduced; the efficiency–depth tension is unprompted, as is the vocabulary of communal learning; and the repertoire of defensive strategies was volunteered within a framed topic.

#### 4.3.1. Positions on “Intellectual Degeneration”, and the Repertoire of Defense

The interview protocol put the proposition to participants directly: “Some argue that over-reliance on AI may cause ‘intellectual degeneration’ in teachers and students. Do you agree?” What follows is therefore a distribution of positions towards a claim we supplied, and not evidence that participants arrived at the concern independently. The 25 cases fell into five groups: explicit endorsement of the degeneration risk—9 (36.0%: C, E, H, K, M, S, U, V, Y); conditionalists, who locate the variable in human agency, self-discipline or attitude—11 (44.0%: A, B, D, F, G, I, L, N, O, P, W); explicit rebuttal—2 (8.0%: J, Q); prolonged ambivalence—1 (4.0%: R); no direct position—2 (8.0%: T, X). For the three cases in which no interview was conducted (D, E, and F), the position was identified in written and recorded materials rather than elicited by this question, and is therefore not strictly comparable with the interview-based classifications.

Participants who endorsed the claim treated degeneration as a substantive risk. Teacher C stated the concern most directly: “To some extent you become… people may get stupider… you just hand everything over to AI; it feels like cutting corners.” Teacher U added: “You no longer think for yourself; you lose your judgment.”

Conditionalists (11, 44.0%) located the decisive factor not in AI itself but in the user: Teacher A—“if everyone has agency (zhutixing), their intelligence cannot decline”; Teacher B—“students who want to think will discipline themselves”; Teacher D—“to do this thing well, not merely to get it done”; Teacher N distinguished “quality dependence” from mindless dependence, insisting that “before, during, and after using a large model, there is always a process of intense human thinking.”

Two participants rejected the claim (2, 8.0%): Teacher J countered with an analogy—“we over-rely on driving… has our instinct for walking degenerated?”; Teacher Q stated, “I don’t think this situation will arise.”

Teacher R represented the ambivalent position and articulated an unresolved cognitive conflict: “I used to grind through dictionaries page by page; now I ask Doubao and instantly know the meaning. Is this efficiency in learning, or have we skipped the learning process and thereby obstructed… mental development? This is a question I keep agonizing over.” She then raised a broader concern: “Will the gap in people’s mental development grow wider and wider in the future?”

Across the accounts, participants described several cognitive defense strategies. Support for the broad category ranges from clear evidence in nine cases to partial evidence in sixteen; the individual strategies below are illustrated by particular cases rather than attributed to every teacher:

Completing work independently before checking. Teacher T: “First put a ‘moral lock’ on students… you must first complete it independently, and then you can check it against what others provide.” Teacher D offered a related formula for creation: “AI does the 0-to-1 for you; you have to do the 2 yourself.”

Retaining the right to make one’s own errors. Teacher L: “If efficiency is the only yardstick, humanity is doomed to lose. But if we change the yardstick to the depth of experience and the internalization of wisdom, things may have a different answer”; “AI optimizes probabilities; humans sediment wisdom”; “machines pursue standardization and denoising… human value may come precisely from our noise.” She therefore called for “daring to take detours personally… keeping a right to be clumsy.”

Requiring cognitive participation. Teacher H emphasized “human-in-the-loop”—“in interacting with AI, the human cannot be absent”; Teacher N: “inject the critical-thinking awareness from the very beginning… if you do not force yourself to think, you easily become dependent on it.”

Avoiding AI deliberately in selected contexts. Teacher Y: “There are some skills I deliberately avoid [teaching]… to prevent them from over-relying on AI.” Teacher R applied a similar boundary to her own child: “If he doesn’t know how to write a character, I actually approve of using Doubao; but when it comes to solving problems or analyzing, I don’t allow it.”

Managing attention. Teacher L extended cognitive defense to attention itself: “In the AI era the scarcest things, besides attention, are judgment”; “the time AI saves you does not become free time. It becomes new goals, new tasks.”

These strategies can themselves be understood as metacognitive acts: while using AI, teachers monitored whether they and their students were still thinking. Agency (zhutixing)—originally a philosophical term—recurred as the concept through which participants qualified their AI use: whether AI causes harm depends, in their accounts, on whether the human retains agency. Teacher S condensed this position into one sentence: “Above AI there is still the human.”

#### 4.3.2. Efficiency Against Depth

Participants widely acknowledged AI’s efficiency gains, yet most also described costs. No protocol item raises an efficiency–depth trade-off, so this material was volunteered. Their accounts revealed several tensions that remained only partially resolved.

The first tension concerned gaining results while losing the learning process. Teacher L captured it through an extended metaphor: “When you throw a complex problem at AI and get a well-structured, logically coherent answer in seconds, the dopamine rush is enormous. But the emptiness that follows is real: I got the result, but I lost the process. I am like a tourist who reaches the summit of Everest by helicopter—I own the view, but not the climber’s muscle memory or the experience of oxygen deprivation.” Teacher X raised the same tension for language learning specifically, asking whether the cycle of trial, error, reflection and internalization still takes place when a machine supplies a standard answer on demand.

The second tension was the coexistence of reduced and increased workload. In contrast to the expectation that AI lightens workload, several teachers reported that AI reduced some tasks while making instructional design more demanding. Teacher S: “If you really want AI well integrated into teaching… in a sense, it has increased our workload.” Teacher R: “Preparing materials no longer costs me time, but instructional design has become harder and more challenging… you feel a kind of racking-your-brains helplessness,” and “this past half year, I have been more tired in class.” Teacher H likewise noted that AI “raises efficiency in some respects while creating new problems in others.”

The third tension concerned foundational training versus rapid results. Teacher R described her approach as “utilitarian”: “In the stage of laying foundations and shaping basic language competence, I think AI should not be used,” yet for graduates about to enter internships she could only choose “getting results fast.” Teacher S, confronted by a doctoral student’s question, “what is the point of repeatedly reading the classics?”, remained, in her words, “unable to let it go even now.”

Participants managed these tensions in two recurring ways. The first was stratified boundary-drawing—deciding when AI should and should not be used—as in Teacher R’s “no AI in the foundation stage” and Teacher S’s “no AI for undergraduates’ homework; clumsy methods in scholarly settings.” The second was to redefine what should count as educational value. Teacher L’s three-tier framework exemplified this approach: “Let AI take over ‘technique’ (shu) and ‘method’ (fa). Hand execution and methodology to it. Keep the ‘Way’ (dao) for yourself”; “Once the ‘heart’ is delegated, we are no longer ourselves.” Her final answer was: “As AI grows stronger, what should we humans grow stronger in? My own answer is inner strength (xinli).” Teacher E located the reconciliation in “implementation”: asking students to build AI’s “perfect” proposals trains their “discernment.” Teacher P described a related change in her view of academic integrity: “Gradually you find that what is produced this way is not yours; it is ultimately not yours, and it will not stay with you.”

#### 4.3.3. Accounts of Cognitive Change

Across disciplines and levels of seniority, participants described elements of a similar trajectory in how they came to understand AI. The “glorification–disenchantment” sequence was especially visible, whereas the form and extent of subsequent “reconstruction” varied. Turning points were usually linked to concrete events. The protocol asked about first contact with AI and so invited a before-and-after account; the three-phase sequence itself, and the vocabulary in which participants describe it, were volunteered. Although some teachers contributed more than one interview, the study did not prospectively track the proposed sequence. As retrospective accounts, they record how participants reconstruct a trajectory when asked, not a sequence the study observed.

Glorification characterized the initial phase. Teacher F wrote: “When I first used it… it was amazing… not a single word could be changed; everything it said seemed right.” Teacher E called DeepSeek’s arrival “overtaking on a curve… it suddenly hit me.”

Disenchantment followed encounters with hallucinations and other failures: Teacher F “later… my understanding gradually rose and I realized what it said was not entirely right—it could be talking nonsense”; Teacher H experienced DeepSeek “from perfect companion to Waterloo”; Teacher X’s turning point was demonstrating in class that “this is not how DeepSeek should be used”; Teacher S summarized AI as “serious-looking nonsense, saying the same thing over and over in circles.”

Reconstruction involved repositioning rather than abandoning AI after disenchantment. Teacher L summarized a year’s journey as “the first stage was breaking the cognitive boundary—from seeing AI as a tool, to a revolution in thinking”; Teacher X moved “from using it as a translation tool” to learning with students and letting AI feed teaching reflection (“on the contrary, it made me reflect on my teaching”); Teacher B described a metacognitive shift—“setting the goal first and then learning creates more miracles than waiting to finish learning before acting”; Teacher R summarized: “The more you use it, the clearer its strengths and weaknesses become”—linking her movement from anxiety to conviction with a more differentiated understanding of the technology.

Many accounts treated disenchantment itself as a source of learning. Teacher S advocated “pouring cold water” in teaching and modeled skepticism for students. Teacher Y’s metaphor was “inviting the wolf into the classroom”—“instead of crying wolf, invite the wolf into the classroom and play together… guidance matters”.

## 5. Discussion

### 5.1. Beliefs and Cognitive Strategies

The accounts suggest a perceived relationship between teachers’ beliefs about AI and the strategies they describe. Instrumental formulations emphasize task-focused use and verification-centered gatekeeping; partnership formulations emphasize sustained dialogue and calibration. These formulations can coexist in the same case, and the later use profiles do not show a simple relationship between partnership language and usage intensity (Section 4.2.2). The interpretation is consistent with Pajares’s (1992) account of beliefs as filters of decision-making and with university teachers’ reported negotiation of GenAI use (Farazouli et al., 2025). Participants also described changes in their orientations, qualifying a view of beliefs as resistant to change (Kagan, 1992). However, the study did not systematically follow those changes as they happened. Correspondence between beliefs and strategies may reflect the coherence of a retrospective account as well as relationships in practice. The evidence supports how participants connect the two in their descriptions, rather than the direction or rate of their influence.

### 5.2. Judgment as an Anchor of Professional Value

Within this sample, teachers shifted the defense of professional value from “what I know” towards “what I can judge”: verification and judgment carry clear evidence in 24 of the 25 cases, and irreplaceability construction appears in 24 of 25. This finding extends Ertmer’s (1999) focus on internal barriers by showing that teachers’ beliefs also concern the source of professional authority. Participants described AI as producing knowledge claims and teachers as evaluating those claims—a redistribution that parallel bodies of literature describe as the renegotiation, rather than the collapse, of epistemic authority (Haase, 2026; Jose et al., 2025; Milella & Cabitza, 2026). The closest analogue lies outside education: newsrooms incorporating generative AI have likewise relocated professional authority from the production of content to its authentication (Ashuri et al., 2026). What distinguishes the present case is that judgment is claimed not only as a competence but as a position in a professional self-description, expressed through labels such as “gatekeeper”, “goalie”, “navigator” and “auditor”. In Biesta et al.’s (2015) terms, these labels name a capacity participants claim they can still exercise under the conditions they face, which is what makes them serviceable for identity work.

### 5.3. Cognitive Defense as Boundary Work

The strategies described in Section 4.3.1—independence first, retaining the right to err, forced thinking, and attention management—can be interpreted as boundary work: participants repeatedly drew a line around “whose cognition” was engaged in a task, seeking to prevent cognitive offloading from becoming cognitive dependence. Comparable boundary work has been documented where professionals must decide how far to let an algorithmic recommendation stand in for their own judgment (Li, 2026). This interpretation is consistent with the cognitive-offloading literature: offloading is not inherently harmful, but its consequences depend on how it is monitored (Risko & Gilbert, 2016). Experimental work raises concerns about reduced regulatory effort in some GenAI-supported tasks (Fan et al., 2025), while observational and review evidence links offloading with variation in critical thinking and learner agency (Gerlich, 2025; G. Wang et al., 2026). These sources do not establish a uniform causal effect of AI use. That these consequences vary across users (Si et al., 2026) lends empirical weight to the conditionalist position articulated by 11 of the 25 teacher cases. The participants’ accounts resembled norms of “monitored offloading” (human-in-the-loop; quality dependence). Their use of agency (zhutixing) to justify these practices also connected cognitive strategy with educational ethics—both a practical stance and a belief summarized as “Above AI there is still the human”.

### 5.4. A Dynamic Model of Belief–Cognition–Practice Interaction

**Figure 1:**
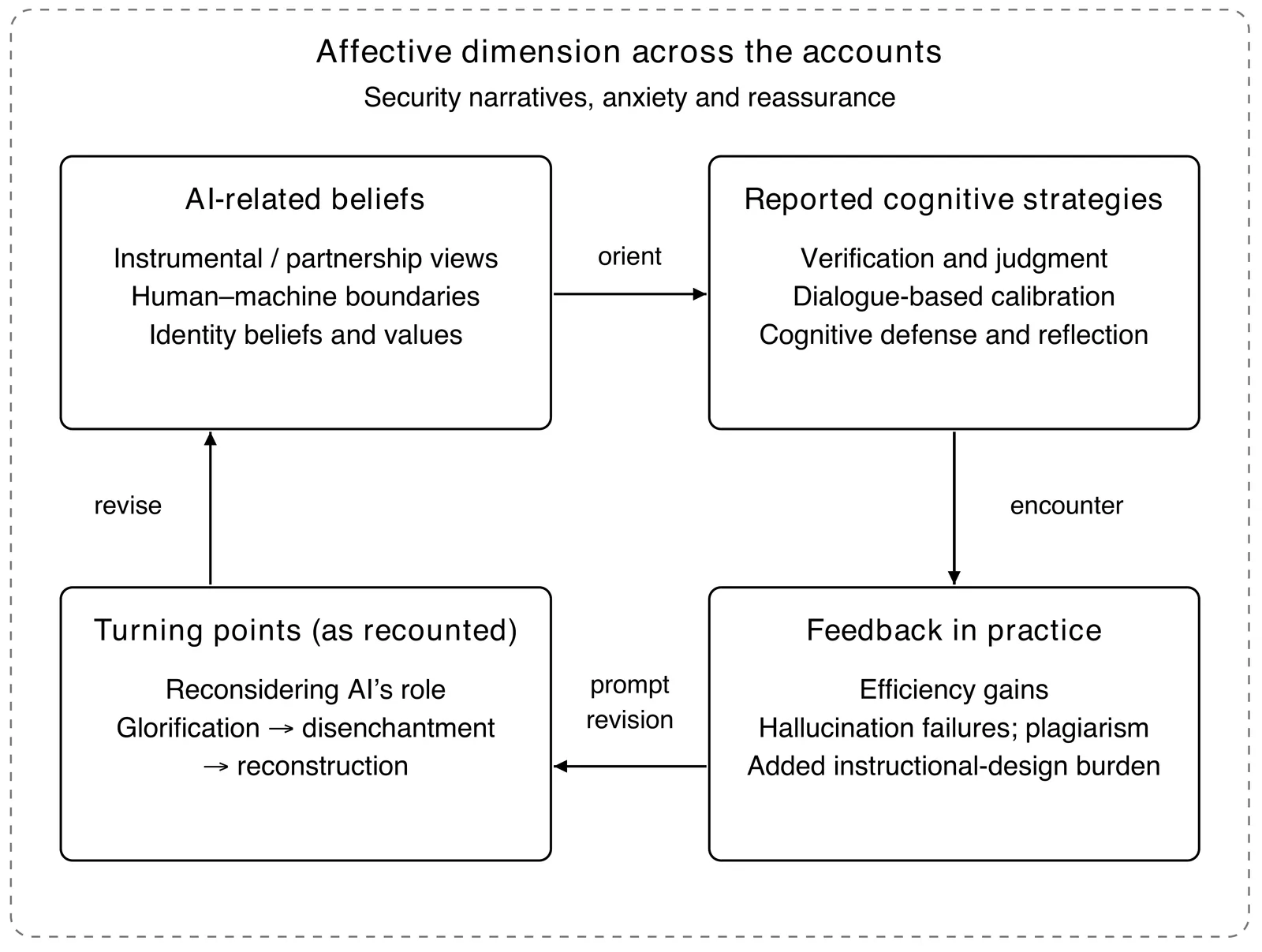
A heuristic interpretation of how belief, cognitive strategy and practice bear on one another in these accounts. Note. The arrows record what participants describe as following from what. They are not tested directions of effect: the accounts are retrospective, cross-sectional and self-reported (Section 5.4).

Bringing together the material in Section 4.2.2 and Section 4.3.3, we set out a heuristic interpretation of how belief, cognition and practice appear to bear on one another in these accounts (Figure 1). Teachers’ AI-related beliefs—instrumental or partnership, boundary beliefs, identity beliefs—appear to orient cognitive strategies such as verification, calibration, defense and reflection. These strategies encounter feedback in practice, including efficiency gains, hallucination-related failures, student plagiarism, and additional workload. Participants’ retrospective accounts locate belief revision at nodes they describe as turning points, after which revised beliefs support new strategies. The security narratives of Section 4.1.3 are interpreted as an affective dimension of this arrangement. We present the figure as an interpretation of retrospective, cross-sectional, self-reported accounts and as a hypothesis for longitudinal research. The arrows record what participants describe as following from what, not tested directions of effect, and nothing in the design permits a claim that one element produces another; establishing that would require following teachers over time and observing what they do rather than what they report.

### 5.5. The Collective Identity Work Under Technological Shock

Taken together, the three themes suggest that these accounts may represent a form of collective identity work rather than isolated individual adjustment. Participants shared a vocabulary (gatekeeping, agency, irreplaceability), a metaphor repertoire (passenger seat, mirror, goalie, filter), and a narrative arc. Membership of a common learning community is a competing explanation for that convergence, not merely a limitation on its generalization: people who talk to one another regularly about a shared problem come to describe it in shared terms, and part of what we report may be the circulation of a vocabulary rather than the independent arrival of 25 teachers at the same position. Three features of the sample bear on how the findings should be taken. Every participant was already engaged with GenAI, which is why disenchantment appears here as a stage within continued use rather than as grounds for abandonment. Foreign-language teachers make up 10 of the 25 cases, which is likely to be one reason the erosion of a disciplinary skill base is so prominent. And the protocol introduced several of the propositions at issue. At the same time, the prominence of “learning from accomplished people” (Teacher V), “teaching is the best learning”, and “mutual inspiration” (Teacher Y) indicates that peer exchange may function as infrastructure for teacher development in the AI era. Comparative work with teachers outside such communities is needed to establish whether these patterns extend further.

### 5.6. What These Accounts Contribute to Research on Intelligence

Research on cognitive offloading examines how external resources change demands on memory, monitoring and task performance (Risko & Gilbert, 2016). The accounts here connect two professional activities: teachers delegate aspects of their own work while also arranging students’ opportunities to undertake or delegate cognitive work. For example, Teacher T describes cross-checking model outputs and asking students to produce an answer before consulting AI. Across the corpus, accounts of efficient preparation sit alongside concerns about preserving students’ engagement. This juxtaposition identifies a tension for research; it does not establish that every teacher reconciles the two activities in the same way.

This perspective suggests a question about the conditions under which offloading supports learning. Studies report both gains in task performance and concerns about regulatory effort, with variation among users and settings (Fan et al., 2025; Gerlich, 2025; Si et al., 2026; G. Wang et al., 2026). Teachers’ task designs may be one contributor to that variation. An assignment that permits a complete AI-generated answer creates different demands from one that asks students to justify an initial answer, compare alternatives or document revision. Here these are implications of reported practice, not measured effects on students.

Two further points follow. The first concerns the lay theories of learning that participants articulate: that difficulty is what makes learning durable, that a fluent answer is a misleading signal of understanding, and that professional expertise requires judgment informed by disciplinary knowledge. Teacher L’s account of owning the view without the climber’s muscle memory is a folk statement of the first; Teacher K’s instruction to refute before examining is a working procedure derived from the second. These propositions are not offered here as correct, and some of them can be pushed too far—difficulty is not valuable in itself, and a belief in necessary struggle can license refusing students tools they would benefit from. They matter because they are reasons teachers give for their task-design choices and may shape the conditions under which students are asked to think.

The accounts also concern the regulation of learning across people. Research on co-regulation and socially shared regulation already examines how others support learners’ planning, monitoring and evaluation, including teachers’ support for these processes (Sulla et al., 2023). We therefore do not propose supervision of another person’s cognition as a new construct. The contribution is a context-specific account of how GenAI-engaged teachers describe regulating their own offloading alongside students’ cognitive participation. Future work could observe whether these reported arrangements are enacted and examine their consequences for students’ monitoring, judgment and learning.

## 6. Conclusions, Implications, and Limitations

Drawing on multi-source qualitative materials from 25 university teachers recruited through an AI-focused learning community and its professional networks, this study examined how they describe their beliefs about GenAI and the cognitive strategies they use alongside it. Four conclusions follow. First, participants described a broadly shared boundary around what AI cannot do—emotion, embodiment, metacognition, tacit knowledge, judgment—which served both to make sense of the technology and to protect a professional position; this boundary was elicited by a direct question and should be taken as the shape of their answers to it. Second, participants commonly reconstructed the teacher role from “knowledge transmitter” towards “guide, designer, companion, gatekeeper”, in terms they volunteered, while ten of the 25 cases (40.0%) retained a reinterpreted claim to expertise. Third, judgment and gatekeeping were re-centered as the defended core of professional work, with verification and judgment carrying clear evidence in 24 of 25 cases. Fourth, participants described managing cognitive participation on both sides of the desk, taking divergent positions on “intellectual degeneration” (9 endorsement, 11 conditional, 2 rebuttal, 1 ambivalent, 2 no position) while describing a varied repertoire of defensive strategies with differing levels of explicit support. The relation between belief and cognition that these accounts suggest is reciprocal but not demonstrated: it is an interpretation of what teachers say, offered for longitudinal and observational examination.

The contribution to research on intelligence lies in connecting teachers’ own cognitive offloading with the learning conditions they report designing for others. Professional judgment and metacognitive regulation are central to these accounts, but their effectiveness requires observational and learning-outcome evidence.

The study carries implications for teachers and institutions. On the one hand, professional development in the AI era could move beyond “learning to use tools” toward “cultivating judgment”, including the disciplinary expertise needed to verify AI output, the metacognitive habit of monitoring cognitive participation, and the willingness “to err personally.” Teacher L’s phrase captures this emphasis: “AI gives the skeleton; the human fills in the soul.” On the other hand, teacher participants reported that AI had not reduced but increased their burden, and described assessment as a domain in which established practices had lost their force without replacements being available. Institutions could respond by supporting instructional-design experimentation, clarifying academic-integrity standards, and creating a fault-tolerant environment in which teachers can test and evaluate new practices, rather than limiting support to tool training.

Regardless, the study is not without limitations. First, the sample was a purposive and snowball sample of teachers already engaged with GenAI, recruited through a common learning community and its professional networks, and the findings cannot be generalized to Chinese university teachers as a whole; the beliefs of resistant and avoidant teachers are not represented, and the community’s shared vocabulary cannot be separated from the convergence we report. Second, the analysis relied on self-reports, so the relation between stated beliefs and classroom practice requires observational evidence. Third, the design is largely cross-sectional and retrospective and cannot establish trajectories or directions of effect. Fourth, broad code definitions and shared interview prompts can increase coverage without establishing independently shared beliefs. The agreement check covers a small sample of meaning units and does not validate all case-level evidence ratings. English translations did not receive an additional bilingual check. Finally, the cases rest on uneven combinations of interviews, self-narratives, published essays, field notes, chat records, product documents and recorded AI dialogues; three cases (D, E, F) rest on documentary material without an interview, and an absent code in a thinly documented case records the limits of the material rather than the absence of a belief.

## Figures and Tables

**Table 1:** Construct assignment of the 20 codes.

Code	Label	Primary Construct	Secondary Component and Boundary Note
B1	Instrumental view of AI	Belief	—
B2	Partnership view	Belief	affect (secondary) where the account is personified
B3	Boundary: emotion and embodiment	Belief	identity (secondary) where used to claim distinctiveness
B4	Boundary: metacognition, tacit knowledge, judgment	Belief	identity (secondary), as B3
B5	AI flaws: hallucination, flattery, formulaicity	Belief	—
B6	“Intellectual degeneration” debate	Belief	—
B7	Survival beliefs: filter, elimination, polarization	Belief	affect (secondary)
B8	Irreplaceability construction	Identity	belief (secondary); coded to identity because the proposition is about the self, not about AI
B9	Teacher role reconstruction	Identity	—
B10	Epistemic authority and expert identity	Identity	belief (secondary)
B11	Teaching and educative beliefs	Belief	—
C1	Mental models and metaphors of AI	Cognition	—
C2	Learning processes	Cognition	practice (secondary), where an activity is described
C3	Verification and judgment strategies	Practice	cognition (inferred); coded to practice because participants report actions, from which strategies are inferred
C4	Metacognition and reflection	Cognition	—
C5	Cognitive defense strategies	Practice	cognition (inferred), as C3
C6	Cognitive conflict and tension	Cognition	affect (secondary)
C7	Cognitive change	Cognition	identity (secondary) where the change is told as self-change
C8	Emotion and security	Affect	belief (secondary)
C9	Communal learning	Practice	—

Assignment rule. Where a segment could be assigned to more than one construct, the primary assignment follows the proposition the participant asserts rather than the vocabulary used to assert it: a statement about what the speaker is (identity) takes precedence over a statement about what AI is (belief); a reported action (practice) takes precedence over the cognitive strategy inferred from it; and an expression of felt state (affect) is recorded as secondary unless the segment asserts nothing else. Secondary components were recorded but do not enter the coverage figures.

**Table 2:** Case profiles (*n* = 25).

Case	Discipline/Role	Years	Institution Type	Tools Most Used	Frequency	Hours/Day	Materials (Transcript Form; Volume)
A	Medical Psychology	13	Chinese medicine	Kimi; Manus; Doubao; Lingguang; Gemini	Almost daily	1	Notes, transcript, narrative (platform-cleaned; 25k)
B	Mental Health Education	8	University for nationalities	ChatGPT; Claude	Every other day	3	Interview, transcript, narrative (platform-cleaned; 18k)
C	College English	24	Provincial key (non-211)	ChatGPT; DeepSeek; Doubao; Coze; Pigai; Xuexitong	Almost daily	1	Interview, transcript (platform-cleaned; 65k)
D	College English	22	Provincial key (non-211)	DeepSeek; ChatGPT; Gemini	Daily	2	Notes, chat records (no interview, documents; 75k)
E	English Major (Teacher Training)	21	Foreign language university	Sider; DeepSeek; WPS AI; Kimi; Doubao; university teaching platform integrated with DeepSeek	Daily	1	Notes, product documents (no interview, documents; 44k)
F	Logistics and Supply Chain Management	24	Normal university	Doubao; Kimi; DeepSeek; ChatGPT; Gemini; Claude; Chaoxing teaching platform with AI functions	Every other day	2	Notes, public essays (no interview, documents; 42k)
G	Law/Political Science	14	211/Double First-Class	ChatGPT; Claude; Get; Ima; Metaso; Google Gemini	Daily	2	Interview, narrative (transcript, form unlabeled; 30k)
H	English Major (Writing)	16	Provincial key (non-211)	Sider; ChatGPT; Kimi	Daily	2	Interview, rubric dialogues (transcript, form unlabeled; 91k)
I	Electronic Commerce	18	Provincial vocational college	Kimi; DeepSeek	Every other day	2	Interview (transcript, form unlabeled; 15k)
J	College English	22	Provincial key (non-211)	DeepSeek; Doubao; Google Translate	Daily	3	Three interviews (both exports; 102k)
K	Information Technology	16	Vocational and technical college	ChatGPT; DeepSeek; Doubao; WPS AI/AI PPT; Coze; Feishu; AMiner	Daily	4	Interview, narrative (verbatim; 29k)
L	Management Science and Engineering	7	Provincial undergraduate	DeepSeek; Sider	Daily	2	Interview, four public essays (platform-cleaned; 85k)
M	Educational Technology	8	Provincial key comprehensive	Doubao; Ima; Sider; Coze Agents; Feishu	Daily	3	Interview, narrative (verbatim; 44k)
N	School of Art and Design, Advertising	10	Science and engineering	Doubao; ChatGPT; Metaso; Feishu; Jimeng	Every other day	2	Interview (platform-cleaned; 23k)
O	Applied Linguistics	30	Sino-foreign cooperative	ChatGPT; Claude; DeepSeek; Kimi	Every other day	2	Interview, public essay (transcript, form unlabeled; 7k)
P	Art Studies	10	Independent undergraduate college	ChatGPT; Gemini; Nano Banana; DeepSeek; Photoshop	Almost daily	3	Interview, narrative (transcript, form unlabeled; 31k)
Q	Journalism	8	Provincial key (non-211)	DeepSeek; ChatGPT	Daily	2	Interview, narrative (platform-cleaned; 32k)
R	English Major (International Communication)	15	Provincial vocational college	Sider; ChatGPT; DeepSeek; Coze	Daily	3	Questionnaire, transcript (transcript, form unlabeled; 29k)
S	Law	8	Political science and law	Doubao; DeepSeek; Ima; Metaso; Bohrium	Weekly	1	Interview, narrative (platform-cleaned; 28k)
T	College English	23	Provincial key (non-211)	Doubao; DeepSeek	Almost daily	2	Transcript (platform-cleaned; 18k)
U	Ideological and Political Education	23	Medical university	Doubao; Zhipu; DeepSeek; WPS AI; BaiduAI	Almost daily	2	Interview (platform-cleaned; 19k)
V	Communication Studies	11	211/Double First-Class	ChatGPT; Claude; Nano Banana Pro; Doubao; Chaoxing Platform	Almost daily	2	Interview, narrative (verbatim; 16k)
W	Art Design	7	Normal university	Kimi; Sider; Stable Diffusion; ChatGPT; Doubao; Sora	Daily	3	Two interviews, narrative (platform-cleaned; 36k)
X	English Major (Medical English)	17	Medical university	DeepSeek; Qwen; iWrite; Hunyuan	Almost daily	2	Interview, transcript (both exports; 127k)
Y	Japanese	19	985/211/Double First-Class	Sider	Almost daily	2	Transcript, notes (verbatim; 36k)

Note. *n* = 25. Discipline, teaching experience, institution type and tools follow the updated participant information sheet. Frequency and hours per day come from the revision-stage follow-up. The fields are descriptive profiles and are not assumed to describe use at the time of each earlier interview. Supplementary Table S1a reports purposes. Twenty-two cases contributed interviews; D, E and F are documentary cases. Transcript labels describe the forms identified in the source inventory; each interview was coded from a single transcript manually checked and corrected against its recording. Additional platform exports were used for source comparison rather than counted as separate cases. Where an export label was not retained, the form is reported as unlabeled. Eleven presentations came from the ten cases marked as having narrative material. Tools are listed as participants named them; version numbers were not recorded.

**Table 3:** Whether the interview protocol introduced each topic.

Code	Label	Status	Protocol Section	Wording That Introduces the Topic
B1	Instrumental view of AI	Framed	5	“automation scenarios”; “teachers’ unique value” invite a tool/partner framing without naming either term
B2	Partnership view	Unprompted	—	no protocol item offers “partner”, “companion,” or “collaborator”; these formulations were volunteered
B3	Boundary—emotion & embodiment	Prompted	6	“what AI cannot replace”
B4	Boundary—metacognition, tacit knowledge, judgment	Prompted	6	“what AI cannot replace”
B5	AI flaws—hallucination, flattery, formulaicity	Framed	4	“verification” invites accounts of failure without naming these three flaws
B6	“Intellectual degeneration” debate	Prompted	4	“Some argue that over-reliance on AI may cause ‘intellectual degeneration’… Do you agree?”—proposition supplied verbatim
B7	Survival beliefs—filter, elimination, polarization	Unprompted	—	no protocol item raises elimination, filtering or polarization; the idiom was volunteered
B8	Irreplaceability construction	Prompted	5	“teachers’ unique value”; §6 “what AI cannot replace”
B9	Teacher role reconstruction	Framed	3	“teaching roles and experiences” and §6 “three identity keywords” ask about role; the specific renamings (guide, designer, gatekeeper, passenger seat) were volunteered
B10	Epistemic authority & expert identity debate	Prompted	5	“the ‘content deliverer’ critique”; “academic authority”
B11	Teaching & educative beliefs	Framed	3	“teaching roles and experiences”
C1	Mental models & metaphors of AI	Unprompted	—	no protocol item asks for a metaphor or model of how AI works
C2	Learning processes	Prompted	2	“prior tools, training, institutional support, technical barriers”
C3	Verification & judgment strategies	Prompted	4	“verification”
C4	Metacognition & reflection	Framed	4	“safeguarding critical thinking” opens the topic; the metacognitive formulations were volunteered
C5	Cognitive defense strategies	Framed	4	“safeguarding critical thinking”; the specific repertoire (independence first, right to err, forced thinking, attention management) was volunteered
C6	Cognitive conflict & tension	Unprompted	—	no protocol item raises an efficiency–depth trade-off
C7	Cognitive change	Framed	1	“first contact with AI” invites a before/after account; the glorification–disenchantment–reconstruction sequence was volunteered
C8	Emotion & security	Framed	5	“automation scenarios” raise replacement; the affective register was volunteered
C9	Communal learning	Unprompted	—	no protocol item asks about the learning community

Note. Prompted = the protocol put the proposition to participants; Framed = the protocol opened the topic without supplying the position or the formulation; Unprompted = no protocol item introduces it. Seven codes are prompted, eight framed and five unprompted. The three document-based cases (D, E, F) were not asked these questions, and their material is not comparable with interview responses on prompted codes (Section 4.3.1).

**Table 4:** Codes qualifying under alternative rules and case definitions.

Rule	*N* = 26	*N* = 25 (Used Here)	*N* = 24
Coverage ≥ 3/4 and clear ≥ 1/3 (the convention used)	17	17	17
Coverage ≥ 4/5 and clear ≥ 1/3	17	17	17
Coverage ≥ 3/4 and clear ≥ 1/2	14	12	14
Coverage ≥ 4/5 and clear ≥ 1/2	14	12	14
Clear evidence ≥ 2/3 of cases	5	6	7

Note. Columns report the number of qualifying codes. *N* = 25 is the analytic corpus; *N* = 26 additionally includes the first author’s reflective material, as in the submitted version. *N* = 24 excludes Teacher O, the case with the smallest recorded volume of material (7k Chinese characters). The same 17 codes qualify under the first two rules across these definitions; stricter rules change the qualifying set. B7 has clear evidence in six cases under each definition and does not meet the stated clear-evidence thresholds.

## Data Availability

The supplementary document contains the reporting codebook, an anonymized code-by-case matrix, participant-use summaries and stance classifications. Appendix D provides aggregate coverage figures.

## Supplementary Materials

The following supporting information can be downloaded at: https://www.mdpi.com/article/10.3390/jintelligence14100254/s1, Section S1: Participant use purposes; Table S1a: Reported purposes and student-related context by case; Table S1b: Descriptive comparison of later usage profiles; Section S2: Case-level evidence matrix; Tables S2a and S2b: Evidence by case; Table S2c: Sensitivity to thresholds and case definitions; Section S3: Reporting codebook; Section S4: Coding agreement; Table S3a: Agreement on primary-code assignment; Table S3b: Disagreement boundaries; Section S5: Stance distributions.

## Appendix A. Abridged Interview Protocol (Six Sections)

(1) Background and first contact with AI; (2) digital literacy and technology adaptation (self-rating 1–10, prior tools, training, institutional support, technical barriers); (3) teaching roles and experiences; (4) digital ethics and critical AI engagement (verification, teaching critical evaluation, ethical dilemmas, safeguarding critical thinking, the “intellectual degeneration” debate); (5) hypothetical and critical thinking (automation scenarios, the “content deliverer” critique, AI-generated assignments, colleagues who refuse AI, teachers’ unique value, academic authority); (6) reflective and future-oriented questions (what AI cannot replace, advice to junior teachers, five-year vision, three identity keywords).

## Appendix B. Codebook Summary

The 20 codes retain their B1–B11 and C1–C9 identifiers for traceability; these historical identifiers do not determine their current construct assignment. Table 1 distinguishes belief, cognition, identity, affect and reported practice. Supplementary Section S3 gives definitions, inclusion/exclusion rules and the boundary refinements recorded in the codebook, together with locations of reporting examples in the manuscript. Primary-code assignment is distinguished from the case-level clear/partial/counter/absent evidence ratings.

## Appendix C. Coding Consistency Check

The first and corresponding authors independently assigned primary codes to 45 meaning units from 19 teacher participants and one first-author reflective source. Agreement was 88.9% (40/45), with *κ* = 0.881. Excluding the first-author unit, S44, gives 39/44 agreements (88.6%) and *κ* = 0.878. The five disagreements concerned adjacent-code boundaries and were reviewed against the original texts, with final adjudication by the corresponding author. The check concerns primary-code assignment, not case-level evidence-strength ratings, and is not a validity test of the interpretation (O’Connor & Joffe, 2020). Supplementary Section S4 reports the calculations and disagreement boundaries.

## Appendix D. Code by Case Matrix: Coverage Summary (*N* = 25)

**Table A1:** Coverage of the 20 codes across the 25 teacher cases.

Code	Clear	Partial	Counter-Case	Coverage	Protocol Status
B1 Instrumental view of AI	12	13	0	25/25	Framed
B2 Partnership view	7	3	0	10/25	Unprompted
B3 Boundary—emotion & embodiment	13	11	1	24/25	Prompted
B4 Boundary—metacognition, tacit knowledge, judgment	14	11	0	25/25	Prompted
B5 AI flaws—hallucination, flattery, formulaicity	10	11	0	21/25	Framed
B6 “Intellectual degeneration” debate	19	6	0	25/25	Prompted
B7 Survival beliefs—filter, elimination, polarization	6	17	0	23/25	Unprompted
B8 Irreplaceability construction	17	7	1	24/25	Prompted
B9 Teacher role reconstruction	25	0	0	25/25	Framed
B10 Epistemic authority & expert identity debate	15	10	0	25/25	Prompted
B11 Teaching & educative beliefs	16	9	0	25/25	Framed
C1 Mental models & metaphors of AI	5	12	0	17/25	Unprompted
C2 Learning processes	21	4	0	25/25	Prompted
C3 Verification & judgment strategies	24	1	0	25/25	Prompted
C4 Metacognition & reflection	13	12	0	25/25	Framed
C5 Cognitive defense strategies	9	16	0	25/25	Framed
C6 Cognitive conflict & tension	17	8	0	25/25	Unprompted
C7 Cognitive change	14	11	0	25/25	Framed
C8 Emotion & security	12	13	0	25/25	Framed
C9 Communal learning	12	13	0	25/25	Unprompted

Note. *N* = 25 teacher cases; the first author’s material is excluded (Section 3.5). Clear, Partial and Counter-Case give the number of cases with clear evidence, partial or indirect evidence, and an explicit counter-case, respectively; Coverage is the number of cases with clear or partial evidence. The only counter-cases were associated with Teacher A (B3 and B8) and are reported in Section 4.1.1 and Section 4.1.3. Protocol status is from Table 3. Coverage is not evidence of convergence on its own and should be taken with the clear-evidence column and the protocol status; several code definitions are broad (Section 3.3). The full per-case matrix is provided in Supplementary Table S2a,b.
