# Supplementary material for: Between Human and Machine: University Teachers’ Accounts of Judgment, Metacognitive Defense, and Cognitive Offloading in Generative AI Use
Source: J Intell. 2026 Oct 10;14(10):254. doi: 10.3390/jintelligence14100254 (PMC13705943; doi:10.3390/jintelligence14100254)
Supplement: Supplementary file 1 [file jintelligence-14-00254-s001.zip › jintelligence-4570238-supplementary.pdf]

Supplementary materials

Between Human and Machine: University Teachers' Accounts of Judgment, Metacognitive Defense, and Cognitive Offloading in Generative AI Use

The supplement provides participant-use purposes, the anonymized case-level matrix, operational code definitions, and coding-agreement details. Case identifiers match the manuscript. The first author's reflective material is excluded from the 25-case matrix. These materials support traceability; they do not turn retrospective accounts into observed cognitive or classroom outcomes.

S1 Participant use purposes

Table S1a summarizes the purposes in the updated participant information sheet used for the revision. These later profiles are not treated as measures of use at the time of every interview. Student-related purpose means an activity described in the profile; it does not establish whether students themselves operated an AI tool. All cases were reported in the follow-up to involve some use in work with students, but that broad designation does not erase differences in purpose.

Table S1a Reported purposes and student-related context by case

Case	Reported purpose	Student-related context in the profile
A	To learn a new skill and support the development of a side business.	Not specified in this profile
B	Support academic research	Not specified in this profile
C	To reduce repetitive teaching work and improve students' language learning through comparison, feedback, and interactive practice	Language learning through comparison, feedback and practice
D	For generating materials and supporting project applications	Materials generation; direct student activity not specified
E	To keep up with technological change; to improve professional efficiency and to develop students' learning capabilities	Developing students' learning capabilities
F	To improve teaching efficiency, enrich case-based instruction; and to cultivate critical thinking and professional judgement	Case-based teaching, critical thinking and professional judgment
G	Support teaching preparation; Save time and extend her capabilities; and Support research writing responsibly	Teaching preparation
H	To improve teaching quality and efficiency while making learning more engaging and personalized	Engaging and personalized learning
I	To improve the depth and efficiency of teaching and learning	Depth and efficiency of teaching and learning
J	Uses AI as a practical teaching assistant to improve instructional efficiency, support students' writing and translation development, provide timely feedback, and make learning more engaging	Writing, translation and timely feedback
K	To improve both quality and efficiency by solving concrete problems in teaching and administrative work.	Teaching problem-solving
L	Using AI was career transformation and personal value creation	Not specified in this profile
M	Uses AI as a creative and efficiency-enhancing tool to make teaching more engaging, personalized, student-centered, and productive, while retaining human expertise and critical judgment.	Engaging, personalized and student-centered teaching
N	To improve efficiency while enhancing teaching quality; and to assist research	Teaching quality

Case	Reported purpose	Student-related context in the profile
O	Uses AI primarily as an “advanced assistant” to improve the efficiency and quality of her teaching and academic work	Teaching and academic work
P	To lower barriers to film production and Improve the whole teaching workflow	Film production and teaching workflow
Q	To make teaching more efficient, accessible, and personalized—while shifting the teacher’s role from routine knowledge delivery toward guidance, discussion, and knowledge creation	Guidance, discussion and knowledge creation
R	For exploring AI was a sense of professional crisis: she feared that AI would threaten language-related employment for both her students and herself	Concern about students’ language-related employment
S	Preparing students for digitalized legal and supervisory practice and teaching innovation.	Preparation for digitalized legal practice
T	Using AI is to treat it as an on-demand assistant and resource bank for teaching, work, and personal learning	Teaching resources
U	To enhance student engagement, understanding, and research efficiency	Student engagement and understanding
V	To use AI as a creative and pedagogical partner that enables more innovative, contextualized, and student-centered teaching	Innovative, contextualized and student-centered teaching
W	To use AI as an assistant that prepares students for an AI-shaped future while improving teaching and creative efficiency	Teaching and preparation for an AI-shaped future
X	She began using AI because she struggled to understand students’ medical papers and needed a practical way to bridge that disciplinary gap	Understanding students’ medical papers
Y	Her initial motivation was personal interest and using AI for research tasks	Not specified in this profile

The comparison in Section 4.2.2 uses B2 coverage (clear or partial) to identify ten partnership-coded cases: A, C, D, H, I, L, N, O, P and Y. B1 has coverage in every case, so these are not mutually exclusive instrumental versus partnership groups. No inferential significance test is applied to this purposive sample.

Table S1b Descriptive comparison of later usage profiles

Profile	B2 clear or partial (n = 10)	Other cases (n = 15)
Hours/day, median (range)	2 (1–3)	2 (1–4)
Daily or almost-daily use	7/10	12/15
Foreign-language/applied-linguistics cases	5/10	5/15

S2 Case-level evidence matrix

C = clear evidence; P = partial or indirect evidence; X = explicit counter-case; 0 = no evidence identified. No evidence is not evidence that a participant lacked a belief. Each row is one teacher case. Coverage is C + P; X is not counted as support. B/C code prefixes preserve historical identifiers rather than define constructs; see manuscript Table 1. The two panels together contain all 25 × 20 cells.

Table S2a Evidence by case

Case	B1	B2	B3	B4	B5	B6	B7	B8	B9	B10	B11
A	P	P	X	P	0	C	0	X	C	P	C
B	P	0	P	C	0	C	P	C	C	C	P
C	C	C	P	C	0	C	P	P	C	C	P
D	P	C	P	C	C	C	P	P	C	C	P
E	P	0	P	C	C	C	0	C	C	C	C
F	C	0	C	P	C	C	C	C	C	P	C
G	P	0	C	C	0	P	C	C	C	P	C
H	C	P	C	C	C	P	P	C	C	C	C
I	P	P	C	C	P	C	C	C	C	C	C
J	P	0	P	P	P	C	C	P	C	C	P
K	C	0	P	P	C	C	P	C	C	P	P
L	P	C	C	C	P	C	P	C	C	C	C
M	C	0	P	C	C	C	P	C	C	C	C
N	C	C	C	P	P	C	P	P	C	P	P
O	C	C	C	P	P	C	P	P	C	P	P
P	C	C	P	C	P	C	P	C	C	C	C
Q	P	0	C	P	C	P	C	P	C	C	P
R	P	0	C	P	P	C	P	C	C	P	C
S	P	0	C	C	C	C	P	C	C	C	C
T	C	0	P	P	C	P	P	P	C	P	P
U	C	0	C	P	P	C	P	C	C	P	C
V	P	0	P	C	P	C	C	C	C	P	C
W	C	0	C	C	P	P	P	C	C	C	C
X	C	0	P	C	C	P	P	C	C	C	C
Y	P	C	C	P	P	C	P	C	C	C	C

Table S2b Evidence by case

Case	C1	C2	C3	C4	C5	C6	C7	C8	C9
A	P	C	P	C	P	P	C	C	C
B	0	C	C	P	P	P	C	C	C
C	P	C	C	C	C	C	P	C	P
D	P	C	C	C	P	C	C	C	P
E	0	C	C	P	P	P	C	P	P
F	P	C	C	C	P	P	C	C	C
G	0	P	C	C	P	C	C	P	C
H	C	C	C	C	C	C	C	P	C
I	P	P	C	P	P	P	P	P	P
J	P	C	C	P	P	C	C	C	P
K	0	C	C	C	P	C	C	P	C
L	C	C	C	C	C	C	C	C	C
M	P	C	C	C	P	C	P	C	P
N	P	C	C	P	C	C	P	P	C
O	0	C	C	P	P	P	P	P	P
P	P	C	C	C	P	C	C	P	P
Q	P	C	C	P	P	C	P	P	C
R	P	C	C	C	C	C	C	C	P
S	C	C	C	C	C	C	P	C	P
T	C	P	C	P	C	P	P	C	P
U	0	P	C	P	P	C	P	P	P
V	0	C	C	P	C	C	C	P	C
W	C	C	C	P	P	C	P	P	P
X	P	C	C	C	P	P	C	P	C
Y	0	C	C	P	C	C	P	C	C

The matrix reproduces the study's saved case-level classifications. The coverage calculations have been recomputed from these cells. This arithmetic check does not independently recode the underlying interviews.

Table S2c Sensitivity to thresholds and case definitions

Rule	N = 26	N = 25	N = 24 excluding O
Coverage ≥ 3/4; clear ≥ 1/3	17	17	17
Coverage ≥ 4/5; clear ≥ 1/3	17	17	17
Coverage ≥ 3/4; clear ≥ 1/2	14	12	14
Coverage ≥ 4/5; clear ≥ 1/2	14	12	14
Clear ≥ 2/3	5	6	7

N = 26 additionally includes the first author's reflective row for comparison with the submitted analysis. N = 24 excludes O, the smallest-volume teacher case (7k Chinese characters). N = 25 is the corpus used in the

manuscript. The three definitions give the same qualifying set under the first two rules; stricter rules are sensitive to the denominator and the case omitted.

S3 Reporting codebook

The definitions below are an English reporting version of the study's codebook. They distinguish primary-code assignment at the meaning-unit level from evidence-strength ratings at the case level. A meaning unit is a sentence or adjacent set of sentences expressing a complete proposition. One primary code is assigned according to the dominant proposition; a secondary code can retain an additional component. Unmatched material is noted for review rather than forced into a category. The B/C identifiers are retained for traceability; construct assignments follow manuscript Table 1.

B1 Instrumental view

Definition: AI as a subordinate tool, assistant, resource bank or mirror.

Include: Claims about service to human purposes, neutrality, or human control. Exclude: Personified partnership (B2), specific output flaws (B5), or distinctively human value (B8).

Reporting examples: manuscript §4.2.2.

B2 Partnership view

Definition: AI as a partner, companion or quasi-person in sustained collaboration.

Include: Personified dialogue, emotional attachment, long-term companionship or collaboration. Exclude: Purely instrumental definitions (B1) or human irreplaceability (B8).

Reporting examples: manuscript §4.2.2.

B3 Boundary involving emotion and embodiment

Definition: A claim that AI lacks human feeling, warmth, bodily experience or embodied understanding.

Include: AI-centered claims about absent affect, empathy or bodily experience. Exclude: Positive claims about teachers' distinctive value (B8) or output errors (B5).

Reporting examples: manuscript §4.1.1.

B4 Boundary involving metacognition and judgment

Definition: A claim that AI lacks reflection, tacit knowledge, intuition or judgment.

Include: Comparisons of what humans and AI can or cannot do. Exclude: Output failures (B5), verification practices (C3), or an account primarily of AI's operation (C1).

Reporting examples: manuscript §4.1.1.

B5 AI flaws

Definition: Negative judgments about hallucination, flattery or formulaic output.

Include: Descriptions of misleading, ingratiating, repetitive or mechanically assembled responses. Exclude: Human–machine capability boundaries (B3/B4) or verification procedures (C3).

Reporting examples: manuscript §4.2.1–4.2.2.

B6 Intellectual degeneration debate

Definition: A position on the proposition that reliance on AI can diminish teachers' or students' cognitive engagement or capabilities.

Include: Endorsement, conditional agreement, rebuttal or ambivalence toward the proposition. Exclude: Specific protective practices (C5) or a general efficiency–depth dilemma not expressing a position on the proposition (C6).

Reporting examples: manuscript §4.3.1.

B7 Survival beliefs

Definition: A broad claim about occupational filtering, elimination or polarization in the AI era.

Include: Survival/filter metaphors, inequalities in adaptation, or claims of technological inevitability. Exclude: Individual anxiety alone (C8) or a specific new teacher role (B9).

Reporting examples: manuscript §4.1.3.

B8 Irreplaceability construction

Definition: A positive claim about the distinctive value of teachers or humans.

Include: Care, aesthetics, judgment, creativity or presence used as grounds for irreplaceability. Exclude: AI-centered deficit claims (B3/B4) or general occupational filtering (B7).

Reporting examples: manuscript §4.1.3.

B9 Teacher role reconstruction

Definition: Explicit naming or description of a reconstructed teacher role.

Include: Guide, designer, coach, companion, gatekeeper, navigator, goalie or comparable role metaphor. Exclude: Claims mainly about knowledge authority (B10) or educational values (B11).

Reporting examples: manuscript §4.1.2.

B10 Epistemic authority and expert identity

Definition: A position on whether the teacher remains a knowledge authority or expert.

Include: Challenges to exclusive authority, retention of expertise or revised expert positioning. Exclude: Naming a new role without an authority claim (B9) or expertise used to verify a specific output (C3).

Reporting examples: manuscript §4.1.2.

B11 Teaching and educative beliefs

Definition: A value claim about the purposes and processes of education.

Include: Educating persons, moral example, learner agency, value formation or the value of effort and reflection. Exclude: A concrete protective task procedure (C5) or role naming as the dominant proposition (B9).

Reporting examples: manuscript §4.1.1–4.1.2.

C1 Mental models and metaphors

Definition: An informal explanation or metaphor for how AI operates.

Include: Probability, corpora, assembly, computation or comparable explanatory models. Exclude: Tool/partner positioning (B1/B2), output flaws (B5), or a human–AI boundary as the dominant claim (B4).

Reporting examples: manuscript §4.2.1.

C2 Learning processes

Definition: An account of learning to use AI and organizing AI-supported work.

Include: Practical experimentation, prompting, defining goals, workflows and learning through doing. Exclude: Output verification (C3), reflective monitoring as the main point (C4), or explicitly social learning (C9).

Reporting examples: manuscript §4.1.2 and 4.3.3.

C3 Verification and judgment strategies

Definition: Reported procedures for evaluating AI outputs.

Include: Cross-model checks, authoritative sources, questioning, knowledge-base constraints, implementation and disciplinary expertise. Exclude: General criticism of AI (B5) or measures primarily protecting students' independent thinking (C5).

Reporting examples: manuscript §4.2.1.

C4 Metacognition and reflection

Definition: Monitoring or evaluating one's own AI use, learning or attention.

Include: Reviewing prompting choices, self-assessment, attention management or reflective adjustment. Exclude: Verification of external output as the main action (C3) or a narrative of belief change (C7).

Reporting examples: manuscript §4.3.1–4.3.3.

C5 Cognitive defense strategies

Definition: Reported actions intended to retain human cognitive participation.

Include: Independent work before comparison, requiring thought, deliberate limits on advanced features or restricting use in foundational learning. Exclude: A stance in the degeneration debate without an action (B6) or output checking alone (C3).

Reporting examples: manuscript §4.3.1.

C6 Cognitive conflict and tension

Definition: An experienced dilemma or unresolved tension in AI use.

Include: Efficiency versus depth, result versus process, reduced versus increased workload, or integrity dilemmas. Exclude: A single stance toward degeneration or occupational survival (B6/B7), or affect without a dilemma (C8).

Reporting examples: manuscript §4.3.2.

C7 Cognitive change

Definition: A retrospective account of changes in understanding or orientation toward AI.

Include: Before/after contrasts, turning points, disenchantment or reorientation. Exclude: Static claims or affect without a change narrative.

Reporting examples: manuscript §4.3.3.

C8 Emotion and security

Definition: An affective account of AI-related professional security or insecurity.

Include: Anxiety, apprehension, reassurance, detachment or pressure to adapt. Exclude: A general elimination thesis (B7) or an unresolved dilemma without an affective focus (C6).

Reporting examples: manuscript §4.1.3.

C9 Communal learning

Definition: Learning about AI through other people.

Include: Peers, experts, students, community exchange, teaching others, workshops or mutual learning. Exclude: Individual study (C2) or a general institutional-support evaluation without a learning process.

Reporting examples: manuscript §4.1.3 and 5.5.

Boundary refinements recorded in V1.1

B9/B11: explicit role naming is B9; an educational value proposition is B11, with a role term retained as secondary where needed. B4/C1: an account primarily of AI's operation is C1; a human–AI capability contrast is B4. B6/C6: an explicit position on the degeneration proposition is B6; a broader unresolved efficiency–depth dilemma is C6. These decisions concern primary-code assignment, not a κ estimate for case-level evidence strength.

Case-level evidence ratings

Clear: an explicit statement supporting the proposition in the participant's own words. Partial: indirect or oblique support. Counter-case: explicit incompatible evidence. No evidence: support not identified in the materials for that case. These ratings are sensitive to source volume, prompting and code breadth. Secondary construct components do not enter coverage totals independently.

S4 Coding agreement

The original exercise assigned one primary code to 45 meaning units. The saved paired records contain 40 agreements. Expected agreement from the marginal code distributions is 0.064691; Cohen's $\kappa = (Po - Pe)/(1 - Pe) = 0.881204$. The source key identifies one unit, S44, as first-author reflective material. Excluding S44 leaves 39 agreements among 44 teacher-derived units; expected agreement is 0.065083 and $\kappa = 0.878453$. These are calculations on primary-code labels, not on clear/partial evidence ratings. The authors confirm that both authors coded and that final adjudication was by the corresponding author.

Table S3a Agreement on primary-code assignment

Sample	Units	Agreements	Agreement	κ
Original consistency exercise	45	40	88.9%	0.881
Teacher-derived units only	44	39	88.6%	0.878

Table S3b Disagreement boundaries

Unit	Coder A	Coder B	Final primary / secondary	Basis of adjudication
S03	B4	C1	B4 / C1	Human–AI capability contrast dominates; probability model retained as secondary.
S13	B2	B9	B9 / B2	Gatekeeper role dominates; personification retained as secondary.
S14	B9	B11	B11 / B9	Educative value dominates; role wording retained as secondary.
S29	B6	C6	B6	Ambivalence expresses a position on the degeneration proposition.
S45	B11	B9	B11	Guidance is an educative belief; no explicit new role is named.

The five disagreements were reviewed against the source materials, with final adjudication by the corresponding author. The small number of units per code does not support stable per-code reliability estimates. Agreement does not establish the validity of the interpretive themes.

S5 Stance distributions

B6 "intellectual degeneration" debate: endorsement of risk 9 (36.0%: C, E, H, K, M, S, U, V, Y; Y with reservations); conditional 11 (44.0%: A, B, D, F, G, I, L, N, O, P, W); rebuttal 2 (8.0%: J, Q); ambivalent 1 (4.0%: R); no position 2 (8.0%: T, X).

Teacher-role positioning: primary self-positioning no longer "knowledge transmitter/knowledge expert" 15 (60.0%: A, B, C, E, F, G, I, L, M, N, O, R, V, W, X); retained a reinterpreted "knowledge expert" positioning 10 (40.0%: D, H, J, K, P, Q, S, T, U, Y).

These stance summaries reproduce the revised manuscript's classifications; they are not estimates of population prevalence. They are distinct from code coverage: evidence that a participant discusses the degeneration proposition does not mean the participant endorses it.
